# Supplementary material for: Exploring the functional meaning of head shape disparity in aquatic snakes
Source: Ecol Evol. 2020 Jul 6;10(14):6993–7005. doi: 10.1002/ece3.6380 (PMC7391336; doi:10.1002/ece3.6380)
Supplement: Supplementary file 1 — Appendix S1 [file ECE3-10-6993-s001.pdf]

Supplementary Material 1: List of scanned specimens per species (N) and their collection number, references for the diet are indicated in the last column. Prey shape is determined by the length/maximal cross-section of the prey: amphibians = bulky, generalist = bulky, fish: depend on the group/species. If several items are present in the diet, the favorite items are indicated by + or ++, and their shape define the "prey shape". If no preference is noted, the shape of the prey item that requires the more extensive manipulation is considered.

Species	N	Specimen number and collection	Diet	Shape	References
Acrochordus granulatus	6	0000.7200, MNHN<br>0000.5196, MNHN<br>0000.7201, MNHN<br>0000.6155, MNHN<br>1900.0356, MNHN<br>1900.0357, MNHN	fish (gobiid)	long	(1–4)
Acrochordus javanicus	5	0000.3294, MNHN<br>MS45, Anthony Herrel<br>MS52, Anthony Herrel<br>0000.5370, MNHN<br>0000.1145, MNHN	fish (+) (eels, catfish)<br>amphibians	long	(4–6)
Afronatrix anoscopus	5	1921.0391, MNHN<br>1916.0215A, MNHN<br>1960.0139, MNHN<br>1943.0079, MNHN<br>1951.0008, MNHN	fish (cyprinid)<br>amphibians (tadpoles ++)	long	(7–9)
Agkistrodon piscivorus	5	0000.4252, MNHN<br>R3979, AMNH<br>R46913, AMNH<br>R50493, AMNH<br>R64620, AMNH	generalist<br>(40% fish)	bulky	(10–14)
Aipysurus fuscus	5	R23488, MCZ<br>R23485, MCZ<br>R23483, MCZ<br>R23482, MCZ<br>R23481, MCZ	fish (labrid and gobiid)	long	(15)
Aipysurus laevis	5	1990.4513, MNHN<br>1990.4507, MNHN<br>1990.4514, MNHN<br>1990.4515, MNHN<br>1999.6566, MNHN	generalist fish (37% Apogonidae, 17% Pempheridae)<br>mollusc (Limidae, Pelecypod	bulky	(15–17)
Atretium schistosum	5	0000.3519, MNHN<br>1946.0064, MNHN<br>0000.7000, MNHN<br>1999.8089, MNHN<br>0000.7414, MNHN	amphibians<br>fish<br>crab?	bulky	(18–20)

Bitia hydroides	7	229793, FMNH<br>229795, FMNH<br>198701, FMNH<br>229791, FMNH<br>211898, CAS<br>211899, CAS<br>211902, CAS	fish (gobiid)	long	(21–24)
Cantoria violacea	7	206912, FMNH<br>250116, FMNH<br>250118, FMNH<br>204970, CAS<br>204971, CAS<br>211909, CAS	crustaceans (shrimps, crabs)	bulky	(21,25,26)
Cerberus rynchops	5	1996.0258, MNHN<br>1900.0417, MNHN<br>1946.0078, MNHN<br>1946.0078A, MNHN<br>1946.0077, MNHN	fish<br>crustaceans	long	(21,25,27,28)
Cylindrophis ruffus	5	0000.3280, MNHN<br>2007.2452, MNHN<br>0000.0440, MNHN<br>0000.3281, MNHN<br>0000.6362, MNHN	fish (eels)<br>snakes<br>caecilians	long	(29–32)
Subsessor bocourti	5	1988.3768, MNHN<br>1970.0556, MNHN<br>1970.0558, MNHN<br>1970.0559, MNHN<br>1885.0333, MNHN	fish (elongated catfish, eels)	long	(21,31,33)
Enhydris chinensis	5	1911.0014, MNHN<br>1911.0015, MNHN<br>0000.8777, MNHN<br>0000.8778, MNHN<br>1906.0217, MNHN	fish (Carassius, Anabas, Cyprinus)<br>amphibians (-)	bulky	(21,25,34–36)
Enhydris enhydris	5	0000.3749, MNHN<br>0000.5567, MNHN<br>1970.0544, MNHN<br>1970.0550, MNHN<br>0000.5528, MNHN	fish (Rasbora, Chandidae, Trichopsis, Trichogaster)	bulky	(21,25,33,37,38)
Ephalophis greyae	5	212348, FMNH<br>212362, FMNH<br>212351, FMNH<br>212361, FMNH<br>212367, FMNH	fish specialist (gobies)	long	(39,40)

Erpeton tentaculatum	5	1970.0564, MNHN<br>1970.0568, MNHN<br>0000.5458, MNHN<br>0000.0924, MNHN<br>0000.0924A, MNHN	fish	long	(21,41)
Erythrolamprus miliaris	5	15426, FMNH<br>15427, FMNH<br>15432, FMNH<br>15433, FMNH<br>217389, FMNH	fish (gobies)<br>amphibians<br>lizards (-)	bulky	(42)
Eunectes murinus	5	0000.7190, MNHN<br>1996.7897, MNHN<br>1996.7898, MNHN<br>1994.1539, MNHN<br>1994.1538, MNHN	generalist (fishes, frogs, turtles, lizards, snakes, caimans, birds, and mammals)	bulky	(42–44)
Farancia erytrogramma	5	1903.0325, MNHN<br>0000.3397, MNHN<br>1991.1666, MNHN<br>0000.3396, MNHN<br>R128620, AMNH	fish (eels)	long	(45–47)
Fordonia leucobalia	6	1974.1331, MNHN<br>1885.0128, MNHN<br>1892.0270, MNHN<br>1885.0545, MNHN<br>217450, FMNH<br>218887, FMNH	crustaceans (dismembered)	bulky	(21,25,38,48–51)
Gerarda prevostiana	4	1946.0079, MNHN<br>1946.0271, MNHN<br>204972, CAS<br>211971, CAS	crustaceans (dismembered)	bulky	(21,25,50–52)
Grayia ornata	5	1996.6644, MNHN<br>1995.9679, MNHN<br>1994.3383, MNHN<br>1994.8079, MNHN<br>1995.9672, MNHN	fish (++) (siluriformes: Clarias, Parauchenolaglis)<br>amphibians	long	(7)
Grayia smithii	5	1998.0603, MNHN<br>1995.3401, MNHN<br>1996.6446, MNHN<br>1994.3393, MNHN<br>1995.3406, MNHN	amphibians (*Xenopus tropicalis* ++, *Ptychadena sp*, tadpoles)<br>fish (siluriforms, cichlids)	bulky	(9,53)
Grayia tholloni	5	1996.6450, MNHN<br>1996.6451, MNHN<br>1988.2341, MNHN<br>1988.2345, MNHN<br>1994.8085, MNHN	fish<br>amphibians	bulky	(7)

Helicops angulatus	5	0000.3609, MNHN<br>0000.1542, MNHN<br>1997.2097, MNHN<br>1997.2032, MNHN<br>1997.2034, MNHN	tadpoles (++) amphibians<br>fish (Astyanax, Copella, Gymnotus, Apistogramma)	long	(42,44,54,55)
Helicops carinicaudus	3	0000.5237, MNHN<br>1887.0447, MNHN<br>87097, CAS	fish (+) (poeciliidae, gobiidae)<br>amphibians	long	(55,56)
Homalopsis buccata	5	1970.0516, MNHN<br>1970.0518, MNHN<br>1974.1333, MNHN<br>1970.0517, MNHN<br>1884.0123, MNHN	fish (tilapia, lebistes, mystus, eels…)<br>amphibians	bulky	(21,24,57–59)
Hydrelaps darwiniensis	5	R86165, AMNH<br>R86166, AMNH<br>R86167, AMNH<br>R86169, AMNH<br>R86172, AMNH	small fish (gobiid)	long	(16,60,61)
Hydrodynastes bicinctus	5	1974.0854, MNHN<br>1889.0398, MNHN<br>1902.0271, MNHN<br>0000.8665, MNHN<br>R88401, AMNH	fish<br>amphibians<br>crustaceans (shrimps)	bulky	(42)
Hydrodynastes gigas	6	1989.3093, MNHN<br>0000.A301, MNHN<br>0000.A302, MNHN<br>1997.2121, MNHN<br>1999.8322, MNHN<br>1997.2347, MNHN	fish<br>amphibians	bulky	(42,62–64)
Hydrophis ornatus	5	0000.0851, MNHN<br>1977.0807, MNHN<br>R66586, AMNH<br>R66588, AMNH<br>R161770, AMNH	fish (Plotosida, Gobiidae)	long	(1,15,16,39)
Hydrophis platurus	5	0000.5137, MNHN<br>1922.0005, MNHN<br>1922.0002, MNHN<br>1994.0659, MNHN<br>1893.0064, MNHN	fish (Clupeidae)	long	(1,16,65,66)
Hydrophis schistosus	5	198586, FMNH<br>202102, FMNH<br>202103, FMNH<br>199488, FMNH<br>218842, FMNH	fish (mainly Ariidae)	long	(1,15,39,67)

Hydrophis spiralis	5	0000.4260A, MNHN<br>0000.4260, MNHN<br>0000.3988, MNHN<br>0000.7723, MNHN<br>R161772, AMNH	fish (+) (Ophichthidae)<br>crustaceans	long	(68)
Hydrophis stokesii	3	212320, FMNH<br>213063, FMNH<br>16774, CAS	fish (Opisthognathidae, Batrachoididae)	long	(15)
Hydrops triangularis	5	1973.0296, MNHN<br>0000.3438, MNHN<br>1978.2500, MNHN<br>1986.0565, MNHN<br>1989.3052, MNHN	fish (+) (Synbranchidae, Gymnotidae)<br>amphibians	long	(42,55,69)
Laticauda colubrina	5	0000.5180, MNHN<br>0000.7702, MNHN<br>0000.5881, MNHN<br>0000.5766, MNHN<br>0000.9053, MNHN	fish (eels)	long	(1,15,70–74)
Lycodonomorphus laevissimus	2	R18223, AMNH<br>156721, CAS	frogs, tadpole<br>fish (Tilapia)	bulky	(75)
Lycodonomorphus rufulus	5	205893, FMNH<br>205889, FMNH<br>0000.3377, MNHN<br>0000.1210, MNHN<br>0000.0563, MNHN	anurans (large tadpoles, frogs)<br>small fish	bulky	(76)
Micrurus lemniscatus	5	1897.0006, MNHN<br>1989.3151, MNHN<br>0000.7658, MNHN<br>1996.7849, MNHN<br>0000.0201, MNHN	fish (eels)<br>snakes, lizards (-)	long	(42,44,54,77)
Micrurus surinamensis	5	1996.7874, MNHN<br>1978.2312, MNHN<br>0000.3926, MNHN<br>1873, Antoine Fouquet<br>1999.8313, MNHN	fish (eels, Gymnotus)	long	(42,44,78)
Myron richardsonii	7	R86236, AMNH<br>R111790, AMNH<br>R111792, AMNH<br>R111793, AMNH<br>114105, CAS<br>135489, CAS<br>135491, CAS	fish (+) (gobiid)<br>nudibranch (-)<br>crabs (-)	long	(15,25)
Naja annulata	5	1967.0455, MNHN<br>1899.0294, MNHN<br>1892.0098, MNHN<br>1967.0452, MNHN<br>0000.8222, MNHN	fish (+) (cichlids of lake Tanganyika....)<br>amphibians	long	(7)

Natriciteres olivacea	5	1896.0518, MNHN<br>0000.6507A, MNHN<br>1896.0520, MNHN<br>0000.6508, MNHN<br>1994.8215, MNHN	frogs<br>small fish	bulky	(8,79,80)
Natrix tessellata	5	2000.5145, MNHN<br>1989.0698, MNHN<br>0000.0641, MNHN<br>1884.0155, MNHN<br>0000.0642, MNHN	fish (+) (Cyprinids: *Gobio gobio*, *Rhodeus sericeus*, *Alburnus alburnus*, and *Pseudorasbora parva*)<br>amphibians	long	(81,82)
Nerodia cyclopion	5	0000.0121, MNHN<br>0000.3482, MNHN<br>1955.0058, MNHN<br>R159217, AMNH<br>R159218, AMNH	fish (sunfish, bass)<br>amphibians	bulky	(83,84)
Nerodia harteri	5	R64408, AMNH<br>R72686, AMNH<br>R72690, AMNH<br>R85314, AMNH<br>R162252, AMNH	fish (Cyprinidae, Itcaluridae…)	long	(83,85–88)
Opisthotropis lateralis	5	R172664, MCZ<br>R172665, MCZ<br>R175987, MCZ<br>R172654, MCZ<br>R172653, MCZ	fish<br>crustacean (freshwater shrimps)	long	(89)
Psammodynastes pictus	6	1891.0077, MNHN<br>1891.0045, MNHN<br>1891.0046, MNHN<br>128402, FMNH<br>148906, FMNH<br>148926, FMNH	small fish<br>anurans (-)<br>crustaceans (prawn)	long	(59)
Pseudoeryx plicatilis	5	0000.3402, MNHN<br>0000.3401, MNHN<br>0000.3401A, MNHN<br>1962.0423, MNHN<br>1978.2550, MNHN	fish (++) (Synbranchus)<br>amphibians	long	(42,54,55,63,90,91)
Pseudoferania polylepis	5	R35067, MCZ<br>R140183, MCZ<br>R129135, MCZ<br>R141689, MCZ<br>1937.0082, MNHN	crustaceans (shrimps Macrobrachium)<br>fish (Megalops, Eleotridae)<br>frogs (-)	long	(21,92)
Regina grahami	5	29565, FMNH<br>30428, FMNH<br>7791, FMNH<br>17033, FMNH<br>17609, FMNH	crayfish (+) (freshly moult crayfish)<br>fish (-)<br>amphibians (-)	bulky	(83,93)

Regina septemvittata	6	3074, FMNH<br>35881, FMNH<br>3076, FMNH<br>3077, FMNH<br>35880, FMNH<br>0000.3492, MNHN	crayfish (freshly moult crayfish)	bulky	(94–96)
Liodytes alleni	5	11047, FMNH<br>22591, FMNH<br>48360, FMNH<br>R159307, AMNH<br>R170180, AMNH	crayfish (hard & soft shell)	bulky	(97–99)
Liodytes rigida	5	0000.1101, MNHN<br>R159322, AMNH<br>R159323, AMNH<br>R160211, AMNH<br>R162319, AMNH	crayfish (hard & soft shell)	bulky	(83,84,99,100)
Liodytes pygaea	5	53688, FMNH<br>53693, FMNH<br>53687, FMNH<br>53691, FMNH<br>95347, FMNH	amphibians (cricket frog, tadpoles, salamander)<br>fish<br>invertebrates (earthworms, leeches)	bulky	(101)
Sinonatrix annularis	5	1902.0080, MNHN<br>1989.0215, MNHN<br>1989.0206, MNHN<br>1999.9017, MNHN<br>1999.9016, MNHN	fish (50%, Misgurnus anguillicaudatus, Channa asiatica)<br>anurans (Rana 50%)	long	(102)
Sinonatrix percarinata	5	1935.0449, MNHN<br>1935.0449A, MNHN<br>1812.0321, MNHN<br>2007.2443, MNHN<br>1812.0319, MNHN	fish (98%, Misgurnus anguillicaudatus, Channa asiatica)<br>anurans (Rana 2%)	long	(102)
Thamnophis atratus	7	R57421, AMNH<br>R162404, AMNH<br>R162405, AMNH<br>212664, CAS<br>212709, CAS<br>212720, CAS<br>220684, CAS	amphibians (frog, toad, larvae, tadpoles, pacific giant salamander larvae)<br>fish	bulky	(19,103–108)
Thamnophis couchii	5	R57423, AMNH<br>R66544, AMNH<br>R108191, AMNH<br>R108192, AMNH<br>R108194, AMNH	fish (salmonids)<br>amphibians (tadpoles, pacific giant salamander larvae)	long	(103,108–113)

Thamnophis rufipunctatus	5	R64376, AMNH<br>R64402, AMNH<br>R68286, AMNH<br>R85996, AMNH<br>R162440, AMNH	fish (green sunfish, rainwbow trout)<br>amphibians (-)	long	(103,114–116)
Xenochrophis piscator	6	1991.1628, MNHN<br>0000.7323, MNHN<br>1991.1627, MNHN<br>1998.8543, MNHN<br>1998.8553, MNHN<br>R34085, AMNH	fish<br>amphibians (toad, frog)<br>rodents (-)	bulky	(31,35,117–121)

References:

1. Glodek GS, Voris HK. Marine snake diets : prey composition , diversity and overlap. Copeia. 1982;1982(3):661–6.

2. Voris HK, Glodek GS. Habitat, diet, and reproduction of the file snake, *Acrochordus granulatus*, in the straits of Malacca. J Herpetol. 1980;14(1):105–8.

3. Lillywhite HB. Husbandry of the little file snake, *Acrochordus granulatus*. Zoo Biol. 1996;15(3):315–27.

4. Lillywhite HB. File snakes (Acrochordidae). In: Hutchins M, Murphy JB, Schlager N, editors. Grzimek's Animal Life Encyclopedia, 2nd Edition, Volume 7, Reptiles. Farmington Hills, MI: Gale Group; 2003. p. 439–44.

5. Dowling HG. The curious feeding habits of the Java Wart Snake. Anim Kingdom. 1960;63:13–5.

6. Boo-Liat L. Notes on the elephant's trunk snake and the puff-faced water snake in Kuala Lumpur. Malayan Nat J. 1964;18:179–83.

7. Pacini N, Harper DM. Tropical Stream Ecology [Internet]. Tropical Stream Ecology. Elsevier; 2008 [cited 2014 Oct 20]. 147–197 p. Available from: http://www.sciencedirect.com/science/article/pii/B978012088449050008X

8. Chippaux J-P. Les serpents d'Afrique occidentale et centrale. Faune et Flore Tropicales. 2006. 1–300 p.

9. Luiselli L. Interspecific relationships between two species of sympatric Afrotropical water snake in relation to a seasonally fluctuating food resource. J Trop Ecol. 2006;22(1):91–100.

10. Vincent SE, Herrel A, Irschick DJ. Sexual dimorphism in head shape and diet in the cottonmouth snake (*Agkistrodon piscivorus*). J Zool. 2004 Sep;264(1):53–9.

11. Lillywhite HB, McCleary RJR. Trophic Ecology of Insular Cottonmouth Snakes: Review and Perspective. South Am J Herpetol. 2008;3(2):175–85.

12. Mitchell J. The Reptiles of Virginia. Washington and London: Smithsonian Institution Press; 1994.

13. Mitchell J. Snakes. Biol Dig. 1991;52(6):17–22.

14. McKnight DT, Harmon JR, McKnight JL, Ligon DB. Notes on the diets of seven sympatric snakes in the genera Agkistrodon, Nerodia, Sistrurus, and Thamnophis. Herpetol Notes. 2014;7(September 2015):171–7.

15. Heatwole H. Sea snakes. Kensington, NSW, Australia, Australia: University of New South Wales Press Ltd; 1987. 148 p.

16. Sherratt E, Rasmussen AR, Sanders KL. Trophic specialization drives morphological evolution in sea snakes. R Soc Open Sci. 2018;5(172141):8.

17. Ineich I, Laboute P. Sea snakes of New Caledonia. IRD Editio. Paris; 2002. 302 p.

18. De Silva A. Atretium schistosum. The IUCN Red List of Threatened Species. Version 2014.2. 2010.

19. Preston DL, Johnson PTJ. Importance of Native Amphibians in the Diet and Distribution of the Aquatic Gartersnake (Thamnophis atratus) in the San Francisco Bay Area of California. J Herpetol. 2012;46(2):221–7.

20. Somaweera R. Sri Lankan Colubrid snakes. Sri Lanka Nat. 2004;5:32–46.

21. Murphy JC. Homalopsid snakes: evolution in the mud. Malabar, Florida: Krieger Publishing Company; 2007.

22. Boulenger GA. Reptilia and Batrachia. The Fauna of British India, including Ceylon and Burma. Kessinger Publishing, LLC; 1890. 564 p.

23. Jayne BC, Ward TJ, Voris HK. Morphology, reproduction, and diet of the marine homalopsine snake *Bitia hydroides* in Peninsular Malaysia. Copeia. 1995;1995(4):800–8.

24. Cantor T. Catalogue of reptiles inhabiting the Malayan peninsula and islands. Calcutta: Printed by J. Thomas; 1847. 182 p.

25. Voris HK, Murphy JC. The prey and predators of Homalopsine snakes. J Nat Hist [Internet]. 2002 Sep [cited 2014 Oct 16];36(13):1621–32. Available from: http://www.tandfonline.com/doi/abs/10.1080/00222930110062642

26. Ghodke S, Chandi M, Patankar V. Yellow-banded Mangrove Snakes (Cantoria violacea) Consume Hard-shelled Orange Signaler Crabs (Metaplax elegans). IRCF Reptil Amphib. 2018;25(1):50–1.

27. Auffenberg W. The Herpetofauna of Komodo, with notes on adjacent areas. Bull Florida State Museum, Biol Sci. 1980;25(2):40–156.

28. Jayne BC, Voris HK, Heang KB. Diet, feeding behavior, growth an numbers of a population of *Cerberus rynchops* (Serpentes: Homalopsinae) in Malaysia. Fieldiana Zoology. Chicago: Field Museum of Natural History; 1988. 36 p.

29. O'Shea M, Halliday T. Reptiles and amphibians. Dorling Kindersley Ltd; 2001. 256 p.

30. Kupfer A, Gower DJ, Himstedt W. Field observations on the predation of the caecilian amphibian, genus Ichthyophis (Fitzinger,1826), by the red-tailed pipe snake *Cylindrophis ruffus* (Laurenti, 1768). Amphibia-Reptilia. 2003;24(June 2001):212–5.

31. Brooks SE, Allison EH, Gill JA, Reynolds JD. Reproductive and Trophic Ecology of an Assemblage of Aquatic and Semi-Aquatic Snakes in Tonle Sap, Cambodia. Copeia [Internet]. 2009;2009(1):7–20. Available from: http://www.bioone.org/doi/abs/10.1643/CE-07-102

32. Greene HW. Dietary correlates of the origin and radiation of snakes. Am Zool. 1983;23(2):431–41.

33. Saint-Girons H, Pfeffer P. Notes sur l'ecologie des serpents du Cambodge. Zool Meded. 1972;47(6):65–87.

34. Mori A. Prey-handling behavior of three species of Homalopsine snakes: features associated with piscivory and Duvernoy's Glands. J Herpetol. 1998;32(1):40–50.

35. Pope CH. Notes on Reptiles from Fukien and other Chinese provinces. American Museum of Natural History; 1929. 153 p.

36. Pope CH. The Reptiles of China. In: Scientific Books: Natural History of Central Asia. 1935. p. 303–4.

37. Karns DR, Murphy JC, Voris HK, Suddeth JS. Comparison of semi-aquatic snake communities associated with the Khorat Basin, Thailand. Nat Hist J Chulalongkorn Univ. 2005;5(October):73–90.

38. Murphy JC, Voris HK, Karns DR, Chan-ard T, Suvunrat K. The Ecology of the Water Snakes of Ban Tha Hin, Songkhla Province, Thailand. Nat Hist Bull Siam Soc. 1999;47(2):129–47.

39. Voris HK, Voris HH. Feeding Strategies in Marine Snakes: An Analysis of Evolutionary, Morphological, Behavioral and Ecological Relationships. Am Zool. 1983;23(2):411–25.

40. Tomascik T, Mah AJ, Nontji A, Mossa MK. The ecology of the Indonesian seas. Oxford University Press; 1997. 656 p.

41. Shaw CE. Tentacled fishing snake. ZooNooz. 1965;38:3–5.

42. Starace F. Guide des serpents et amphisbènes de Guyane. Ibis Rouge. 1998. 452 p.

43. Campos VA, Oda FH, Custódio RJ, Felismino MF. *Eunectes murinus* (Green Anaconda). Diet. Herpetol Rev. 2011;42(1):99–99.

44. Martins M, Oliveira ME. Natural History of Snakes in Forests of the Manaus Region, Central Amazonia, Brazil. Herpetol Nat Hist. 1998;6(2):78–150.

45. Haltom WL. Alabama reptiles, (Alabama museum of natural history. Museum paper). University. 1931. 145 p.

46. Cochran TT. A review and Synthesis of existing literature on rainbow snakes, *Farancia erytrogramma*. Bull Chicago Herpetol Soc. 2011;46(12):157–61.

47. Richmond ND. The habits of the rainbow snake in Virginia. Copeia. 1945;1945(1):28–30.

48. Günther ACLG. The Reptiles of British India. Ray Societ. Hardwicke R, editor. London; 1864. 540 p.

49. Gow G. Graeme Gow's Complete Guide to Australian Snakes. Harpercollins; 1991. 181 p.

50. Karns DR, Voris HK, Goodwin TG. Ecology of oriental-australian rear-fanged water snakes (Colubridae: Homalopsinae) in the Paris Ris Park Mangrove Forest, Singapore. Raffles Bull Zool. 2002;50(2):487–98.

51. Jayne BC, Voris HK, Ng PKL. How big is too big? Using crustacean-eating snakes (Homalopsidae) to test how anatomy and behaviour affect prey size and feeding performance. Biol J Linn Soc. 2018;123(3):636–50.

52. Jayne BC, Voris HK, Ng PKL. Snake circumvents constraints on prey size. Nature. 2002;418(6894):143.

53. Pauwels OSG, Lenglet G, Trape J-F, Dubois A. *Grayia smithii* (Leach, 1818). Smith's African Water Snake. Diet. African Herp News. 2000;31 October:7–9.

54. Dixon JR, Soini P. The reptiles of the Upper Amazon Basin, Iquitos region. Milwaukee, Wisconsin, USA: Milwaukee Public Museum; 1986. 154 p.

55. Scartozzoni RR. Estratégias reprodutivas e ecologia alimentar de serpentes aquáticas da tribo Hydropsini (Dipsadidae, Xenodontinae). 2009;

56. Marques OA V., Sazima I. História natural dos répteis da estação ecológica Juréia-Itatins. In: Marques OA V., Duleba W, editors. Estação Ecológica Juréia-Itatins: Ambiente Físico, Flora e Fauna Holos. Holos. Holos, Ribeirão Preto; 2004. p. 257–77.

57. Berry PY, Lim GS. The breeding pattern of the puff-faced water snake, *Homalopsis buccata* Boulenger. Copeia. 1967;1967(2):307–13.

58. van Hoesel JKP. Ophidia Javanica. Bogor, Indonesia: Museum Zoologicum Bogoriense; 1959.

59. Tweedie MWF. The snakes of Malaya. Singapore: Government Printing Office; 1953. 139 p.

60. Ehmann H. Reptiles. In: Encyclopedia of Australian animals. Angus & Ro. Pymble, N. S. W.; 1992.

61. Guinea ML, McGrath P, Love B. Observations of the Port Darwin sea snake *Hydrelaps darwiniensis*. North Territ Nat. 1993;14:28–30.

62. Giraudo AR, López MS. Diet of the large water snake Hydrodynastes gigas (Colubridae) from northeast Argentina. Amphibia-Reptilia. 2004;25(2):178–84.

63. Strussmann C, Sazima I. The snake assemblage of the Pantanal at Pocone, Western Brazil: faunal composition and ecological summary. Stud Neotrop Fauna Environ. 1993;28(3):157–68.

64. Strussmann C, Sazima I. Esquadrinhar com a cauda: uma tatica de caca da serpente Hydrodynastes gigas no Pantanal, Mato Grosso. Memorias do Inst Butantan (Sao Paulo). 1990;52(2):57–61.

65. Murphy JB, Schlager N. Grzimek's Animal Life Encyclopedia. 2003.

66. Kropach CN. The yellow-bellied sea snake, Pelamis, in the eastern Pacific. In: Dunson WA, editor. The biology of sea snakes. Baltimore: University Park Press; 1975. p. 185–213.

67. Voris HK, Voris HH, Liat LB. The food and feeding behavior of a marine snake, Enhydrina schistosa (Hydrophiidae). Copeia. 1978;1978(1):134–46.

68. Karthikeyan R, Balasubramanian T. Species diversity of sea snakes (Hydrophiidae) distributed in the Coramantal Coast (East coast of India). Int J Zool Res. 2007;3(3):107–31.

69. de Albuquerque NR, Camargo M. Hábitos alimentares e comentários sobre a predação e reprodução das espécies do gênero Hydrops Wagler, 1830 (Serpentes : Colubridae). Comun do Mus Ciências e Tecnol da PUCRS. 2004;1:21–32.

70. Gorman GP, Licht P, McCollum F. Annual Reproductive Patterns in Three Species of Marine Snakes from the Central Philippines. J Herpetol. 1981;15(3):335–54.

71. Shine R, Reed RR, Shetty S, Cogger HG. Relationships between sexual dimorphism and niche partitioning within a clade of sea-snakes (Laticaudinae). Oecologia. 2002;133(2002):45–53.

72. Ineich I, Bonnet X, Brischoux F, Kulbicki M, Seret B, Shine R. Anguilliform fishes and sea kraits: neglected predators in coral-reef ecosystems. Mar Biol. 2007;51(2):793–802.

73. Shetty S, Shine R. Sexual divergence in diets and morphology in Fijian sea snakes Laticauda colubrina (Laticaudinae). Austral Ecol. 2002;27(1):77–84.

74. Voris HK. The role of sea snakes (Hydrophiidae) in the trophic structure of coastal ocean communities. J Mar Biol Assoc India. 1972;14(2):429–42.

75. Haagner G V., Branch WR. A taxonomic revision of the dusky-bellied water snake, Lycodonomorphus laevissimus Serpentes: Colubridae. J African Zool. 1994;237–50.

76. Taylor P. An observation on the feeding habits of *Lycodonomorphus rufulus*. J Herpetol Assoc Africa [Internet]. 1970;6(1):19–20. Available from: http://www.tandfonline.com/doi/abs/10.1080/04416651.1970.9650767

77. Sazima I, Abe SA. Habits of five Brazilian snakes with coral-snake pattern, including a summary of defensive tactics. Stud Neotrop Fauna Environ. 1991;26(3):159–64.

78. Cunha OR, Nascimiento FP. Ofídios da Amazônia. As cobras da região Leste do Pará. Bol do Mus Para Hist Nat e Ethnogr. 1993;9(1):1–191.

79. Razzetti E, Msuya CA. Field Guide to the amphiians and reptiles of Arusha National Park (Tanzania). Varese, Italy: Edizioni Negri and Istituto OIKOS; 2002. 84 p.

80. Rödel M-O, Spawls S. *Natriciteres olivacea*. IUCN Red List Threat Species Version 20142. 2010;

81. Filippi E, Capula M, Luiselli L, Agrimi U. The prey spectrum of *Natrix natrix* (Linnaeus, 1758) and *Natrix tessellata* (Laurenti, 1768) in sympatric populations. Herpetozoa. 1996;8(3/4):155–64.

82. Luiselli L, Capizzi D, Filippi E, Anibaldi C, Rugiero L, Capula M. Comparative diets of three populations of an aquatic snake (*Natrix tessellata*, Colubridae) from Mediterranean streams with different hydric regimes. Copeia. 2007;2007(2):426–35.

83. Gibbons JW, Dorcas ME. North American watersnakes, a natural history. Animal Nat. University of Oklahoma Press; 2004. 496 p.

84. Kofron CP. Foods and Habitats of Aquatic Snakes (Reptilia, Serpentes) in a Louisiana Swamp. J Herpetol. 1978;12(4):543–54.

85. Rose F. Aspects of the biology of the Concho watersnake (Nerodia harteri paucimaculata). Texas J Sci. 1989;41:115–30.

86. Dorcas ME, Mendelson JR. Distributional notes on *Nerodia harteri harteri* in Parker and Palo Pinto counties, Texas. Herpetol Rev. 1991;22:117–8.

87. Greene BD, Dixon JR, Mueller JM, Whiting MJ, Thornton OW, Thornton OWJ. Feeding ecology of the Concho water snake, *Nerodia harteri paucimaculata*. J Herpetol. 1994;28(2):165–72.

88. Ernst CH, Ernst EM. Snakes of the United States and Canada. Smithsonian Books; 2003. 680 p.

89. Wang Y, Lau M. *Opisthotropis lateralis*. IUCN Red List Threat Species 2012 eT192152A2047730. 2012;

90. Carvalho MA, Nogueira F. Serpentes da área urbana de Cuiabá, Mato Grosso: aspectos ecológicos e acidentes ofídicos associados. Cad Saude Publica. 1998;14(4):753–63.

91. Kaefer IL, Montanarin A. Pseudoeryx plicatilis (South American Pond Snake). Diet. Herpetol Rev. 2010;41(3):372.

92. Shine R. Strangers in a Strange Land : Ecology of the Australian Colubrid Snakes. Copeia. 1991;1991(1):120–31.

93. Hall RJ. Ecological observations on Graham's water snake, Regina grahami (Baird and Girard).

Am Midl Nat. 1969;81(1):156–63.

94. Godley JS, McDiarmid RW, Rojas NN. Estimating prey size and number in crayfish-eating snakes, genus Regina. Herpetologica. 1984;40(1):82–8.

95. Branson BA, Baker EC. An ecological study of the Queen snake, Regina septemvittata in Kentucky. Tulane Stud Zool Bot. 1974;18(January):153–71.

96. Wood JT. Observations on *Natrix septemvittata* (say) in Southwestern Ohio. Am Midl Nat. 1949;42(3):744–50.

97. Dwyer CM, Kaiser H. Relationship between skull form and prey selection in the Thamnophiine snake Genera Nerodia and Regina. J Herpetol. 1997;31(4):463–75.

98. Franz R. Observations on the food, feeding behavior, and parasites of the striped swamp snake, Regina alleni. Herpetologica. 1977;33(1):91–4.

99. Godley JS. Foraging Ecology of the Striped Swamp Snake, Regina alleni, in Southern Florida. Ecol Monogr. 1980;50(4):411–36.

100. Durso AM, Willson JD, Winne CT. Habitat influences diet overlap in aquatic snake assemblages. J Zool. 2013;291(3):185–93.

101. Palmer WM, Paul JR. The black swamp snake, *Seminatrix pygaea paludis* Dowling, in North Carolina. Herpetologica. 1963;19(3):219–21.

102. Mao J-J. Population ecology of genus Sinonatrix in Taiwan. Trier; 2003.

103. Rossman DA, Ford NB, Seigel RA. The Garter Snakes: Evolution and Ecology. Animal Nat. University of Oklahoma Press; 1996. 336 p.

104. Fitch HS. A biogeographical study of the ordinoides artenkreis of garter snakes (genus Thamnophis). Berkley an. Vol. 44. University of California publications in zoology; 1940. 149 p.

105. Fox WR. Relationships Among the Garter Snakes of the Thamnophis Elegans Rassenkreis. University of California Press; 1951. 485–529 p.

106. Fitch HS. The feeding habits of California garter snakes. Calif Fish Game. 1941;27:2–32.

107. Lind AJ, Welsh HHJ. Ontogenetic changes in foraging behaviour and habitat use by the Oregon garter snake , Thamnophis atratus hydrophilus. Anim Behav. 1994;48:1261–73.

108. Edgehouse MJ. Garter Snake (Thamnophis) Natural History : Food Habits and Interspecific Aggression. 2008.

109. Fitch HS. Study of Snake Populations in Central California. Am Midl Nat. 1949;41(4):513–79.

110. Drummond HM. Aquatic foraging in garter snakes : a comparison of specialists and generalists. Behavior. 1983;86(1):1–30.

111. Lind AJ. Ontogenetic Changes in the Foraging Behavior, Habitat Use and Food Habits of the Western Aquatic Garter Snake, Thamnophis couchii, at Hurdygurdy Creek, Del Norte County, California. 1990.

112. Alfaro ME. Forward attack modes of aquatic feeding garter snakes. Funct Ecol. 2002 Apr;16(2):204–15.

113. Drummond HM. The role of vision in the predatory behaviour of natricine snakes. Anim Behav. 1985;33:206–15.

114. Fleharty L, Fleharty ED. Comparative Ecology of Thamnophis elegans, T. cyrtopsis, and T. rufipunctatus in New Mexico. Southwest Nat. 1967;12(3):207–29.

115. Rosen PC, Schwalbe CR. Status of the Mexican and narrow-headed gartersnakes (Thamnophis eques megalops and Thamnophis rufipunctatus rufipunctatus) in Arizona. Albuquerque, New Mexico; 1988.

116. Stebbins RC. A Field Guide to Western Reptiles and Amphibians. Peterson F. Boston, Massachusetts: Houghton Mifflin Harcourt; 2003. 560 p.

117. Das I. A photographic guide to snakes and other reptiles of India. London, United Kingdom: New Holland Publishers Ltd; 2002. 144 p.

118. De Silva A, Das I. A Photographic Guide To Snakes & Other Reptiles Of Sri Lanka. Photograph. New Holland Publishers Ltd; 2004. 144 p.

119. Sharma S. Group hunting and mass feeding by checkered keel-back water snake (Xenochrophis piscator) in Shipra River. Anņe Biol South Asian Reptil Netw. 2004;10.

120. Al Moktadir N, Hasan MK. Unusual feeding behavior of the Checkered Keelback Xenochrophis piscator on Jahangirnagar University Campus, Savar, Dhaka, Bangladesh. Reptil RAP [Internet]. 2016;18:32–3. Available from: www.zoosprint.org/Newsletters/ReptileRap.htm

121. Hossain ML. Food habits of checkered keelback, Xenochrophis piscator (Schneider, 1799), in Bangladesh. Bangladesh J Zool. 2016;44(1):153–61.
