# Supplementary material for: Exploring the functional meaning of head shape disparity in aquatic snakes
Source: Ecol Evol. 2020 Jul 6;10(14):6993–7005. doi: 10.1002/ece3.6380 (PMC7391336; doi:10.1002/ece3.6380)

Supplementary Material 2: Assessment of the error in landmark positioning using a principal component analysis. Landmarks were placed ten times on three different specimens of the same species. The principal component plot shows that variation due to the placement of the landmarks is lower than variation among individuals.

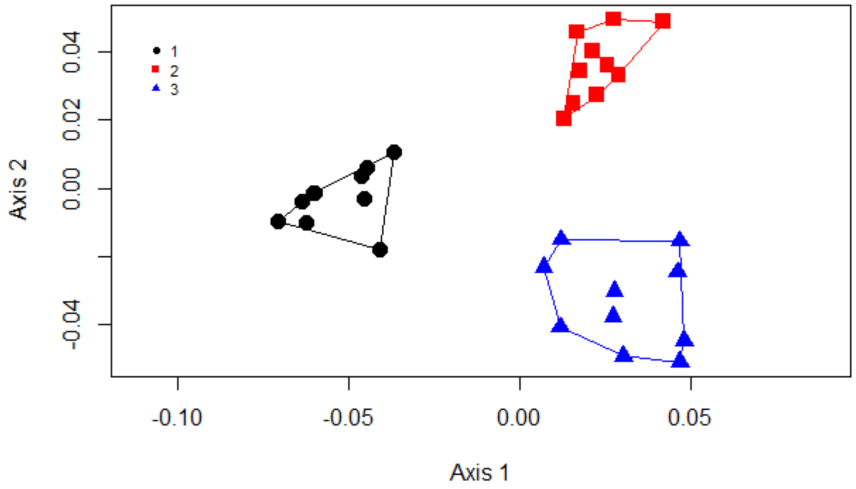

Supplement: Supplementary file 2 — Appendix S2 [file ECE3-10-6993-s002.pdf]
