# Supplementary material for: Exploring the functional meaning of head shape disparity in aquatic snakes
Source: Ecol Evol. 2020 Jul 6;10(14):6993–7005. doi: 10.1002/ece3.6380 (PMC7391336; doi:10.1002/ece3.6380)
Supplement: Supplementary file 4 — Appendix S4 [file ECE3-10-6993-s004.pdf]

Supplementary Material 4: Gape angles in aquatically foraging snakes.

- *Thamnophis couchii* from Supplementary Movie in (Alfaro, 2002)

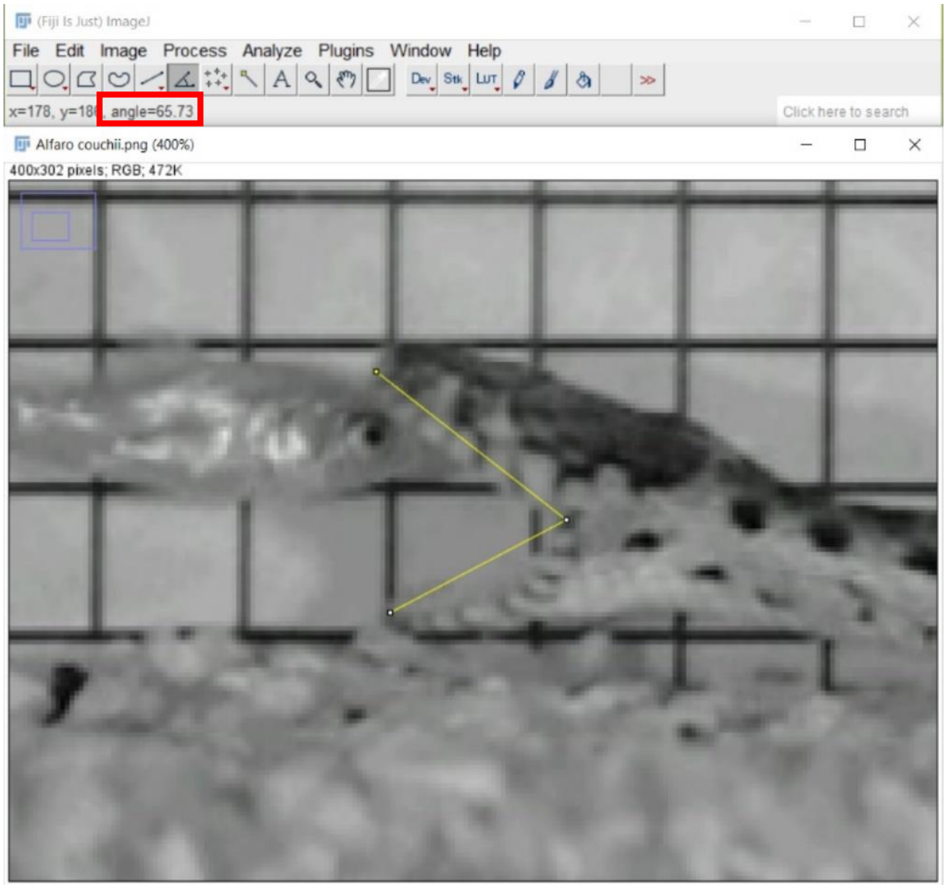

- *Natrix tessellata* (Natricinae)

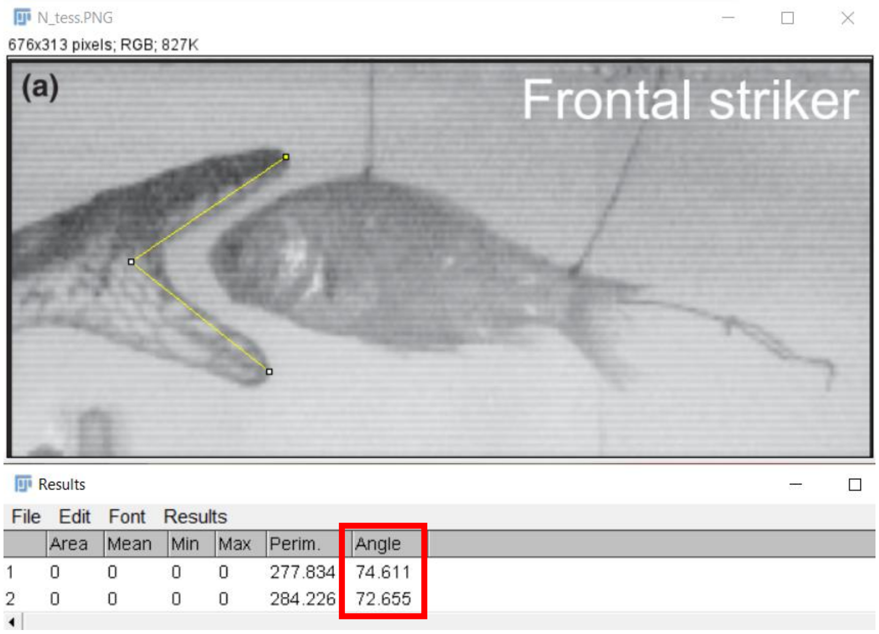

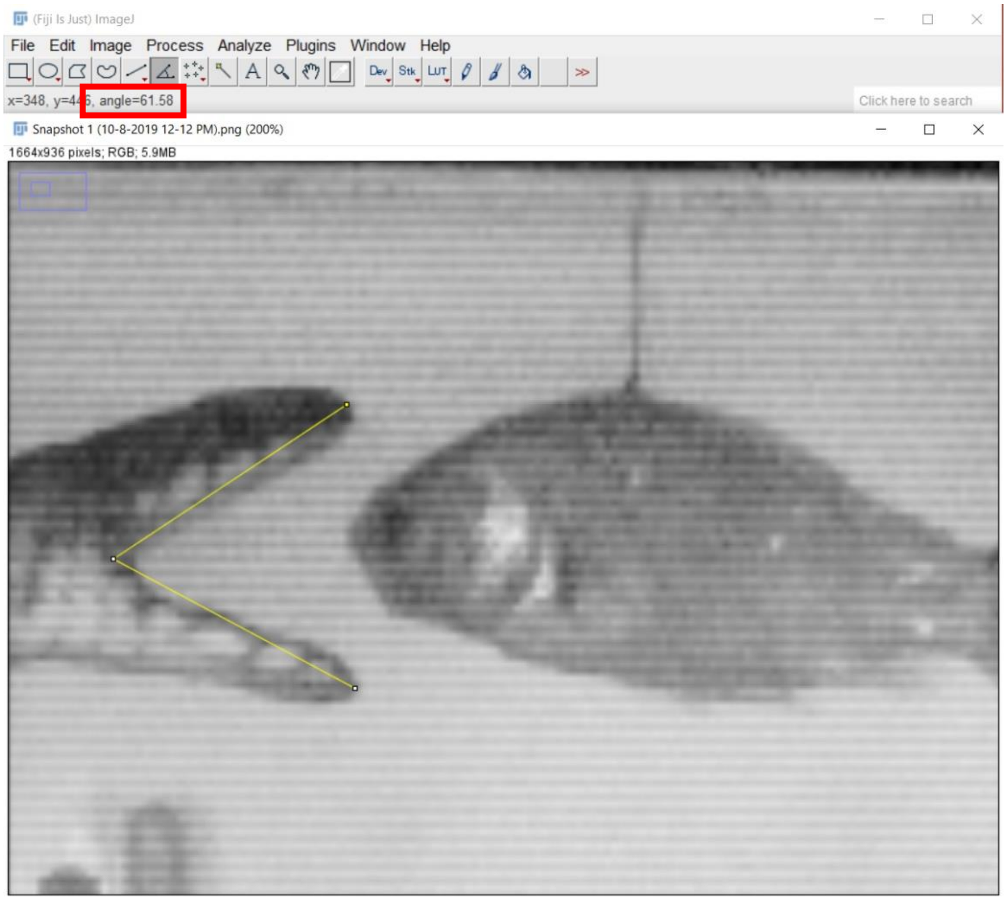

Both images from (Herrel *et al.*, 2008)

- *Homalopsis buccata* (Homalopsidae)

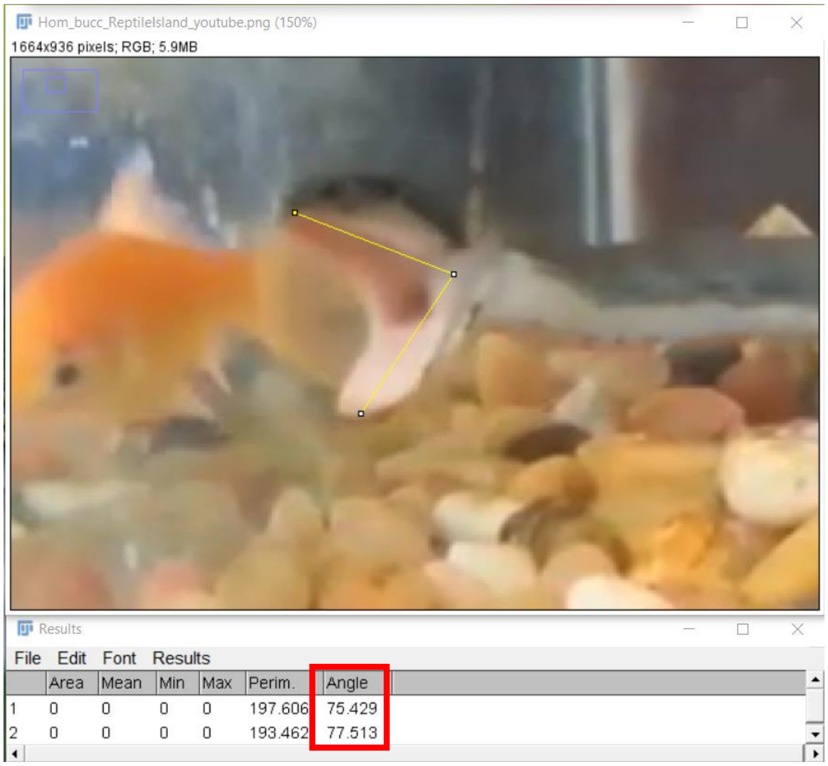

Image from Reptile Island, youtube video. Gape angle might be overestimated due to the camera angle.

- *Subsessor bocourti* (Homalopsidae)

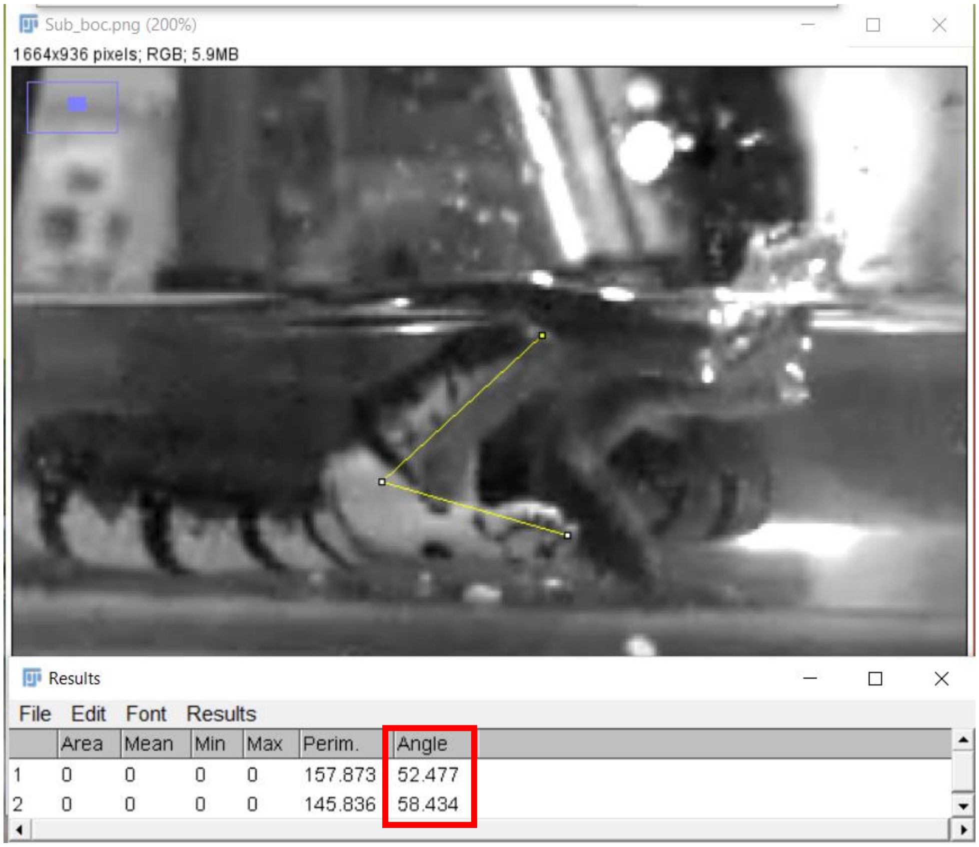

Image from our unpublished data recorded with a high-speed Miro Phantom camera.

References

Alfaro, M.E. 2002. Forward attack modes of aquatic feeding garter snakes. *Funct. Ecol.* **16**: 204–215.

Herrel, A., Vincent, S.E., Alfaro, M.E., Van Wassenbergh, S., Vanhooydonck, B. & Irschick, D.J. 2008. Morphological convergence as a consequence of extreme functional demands: examples from the feeding system of natricine snakes. *J. Evol. Biol.* **21**: 1438–1448.
