# Supplementary material for: Exploring the functional meaning of head shape disparity in aquatic snakes
Source: Ecol Evol. 2020 Jul 6;10(14):6993–7005. doi: 10.1002/ece3.6380 (PMC7391336; doi:10.1002/ece3.6380)
Supplement: Supplementary file 5 — Appendix S5 [file ECE3-10-6993-s005.pdf]

Supplementary Material 5: Rotation process on Blender™

Screenshots of the superimposed skull and jaw parts of two models. The Generalized Procrustes Analysis allows the models to be aligned and scaled making this process homologous between the different shapes. The following screenshots show the two parts that are computationally rotated: the skull part and the jaw.

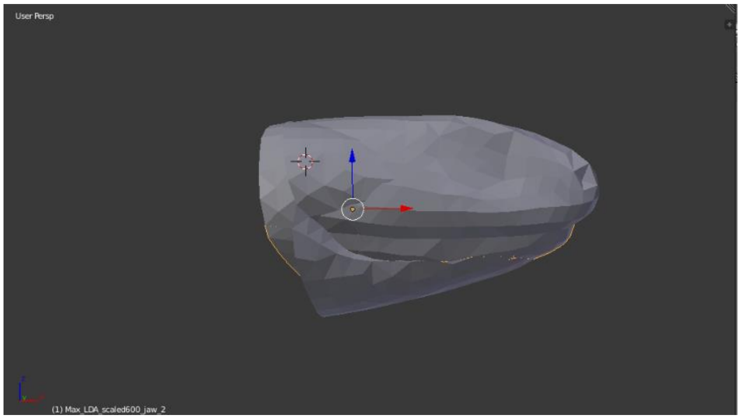

The first screenshot shows two superimposed models before the opening process in side view.

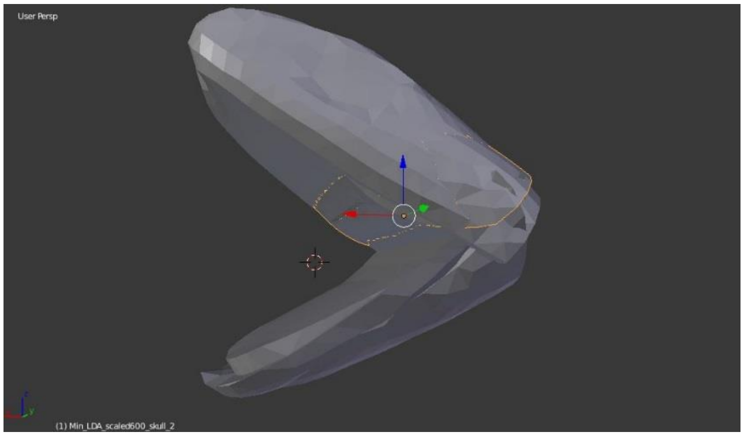

The second screenshot shows two models after opening the mouth to an angle of 70°.

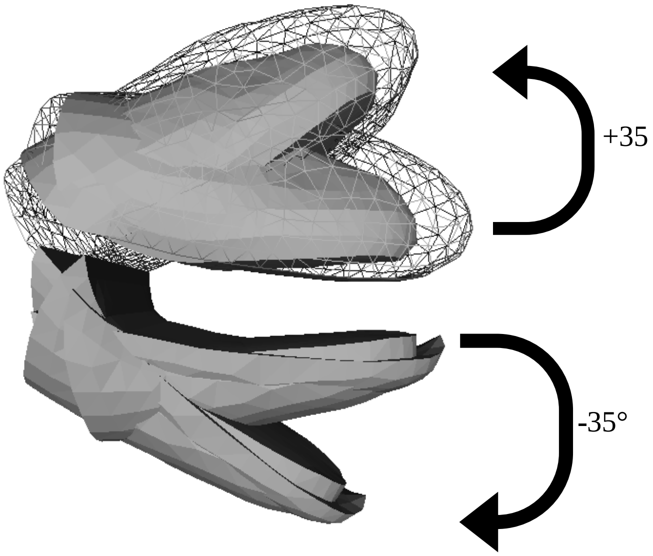

Skull parts of the models viewed from the side before and after rotation of +35° in Blender™. One of the model appears wire-like to show the homology of the process.

Jaw parts of two models viewed from the side before and after rotation of -35° in Blender™.
