# Supplementary material for: Exploring the functional meaning of head shape disparity in aquatic snakes
Source: Ecol Evol. 2020 Jul 6;10(14):6993–7005. doi: 10.1002/ece3.6380 (PMC7391336; doi:10.1002/ece3.6380)
Supplement: Supplementary file 7 — Appendix S7 [file ECE3-10-6993-s007.pdf]

Supplementary Material 7: Steady drag ($2F_d/\rho S$ of Eq (3)) depending on the squared velocity (U^2) of each strike for the five head models tested. Linear regression lines are drawn using dashed lines, the regression coefficients (y) correspond to the drag coefficient (C_d) of each shape and are indicated in the table below the graph. To compare with previous work (Segall *et al.*, 2019), the drag coefficients associated with the mean head shape of non-aquatically (orange line) and aquatically (dark blue line) foraging snakes have been added using solid lines.

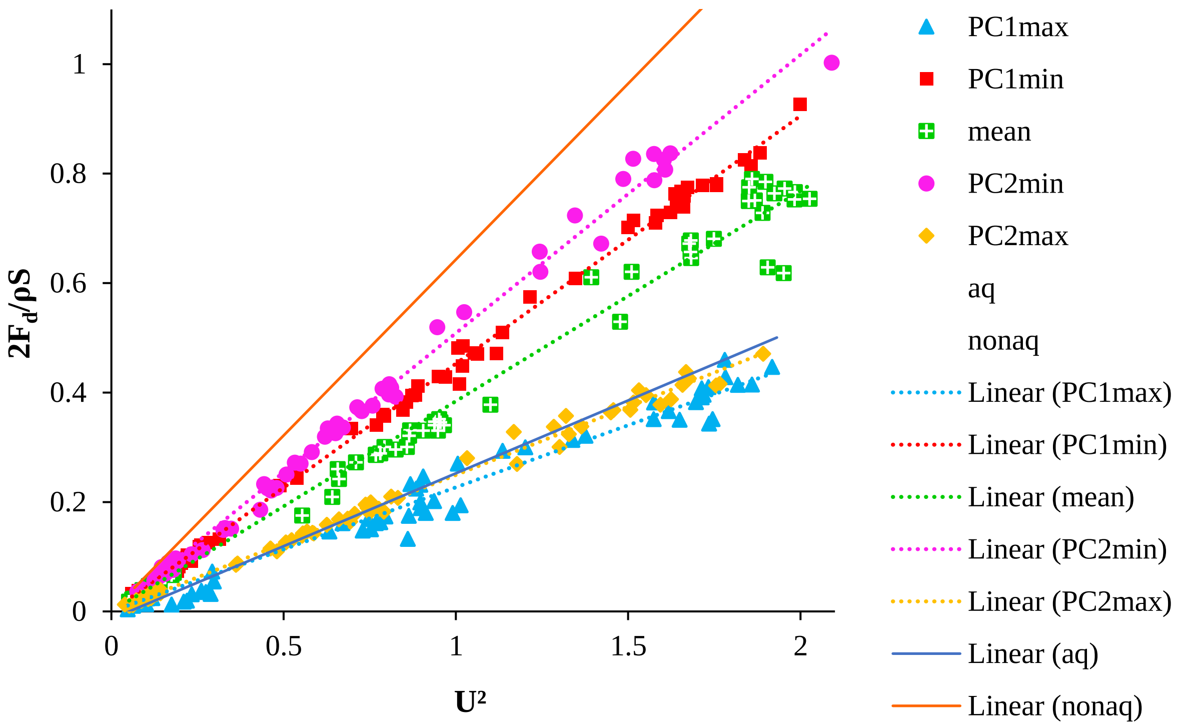

Model	C_d	R^2	N
PC1max	0.2268	0.9737	61
PC1min	0.4527	0.9977	66
Mean	0.3842	0.9865	63
PC2min	0.5086	0.9946	67
PC2max	0.2494	0.9943	70
