# Supplementary material for: Exploring the functional meaning of head shape disparity in aquatic snakes
Source: Ecol Evol. 2020 Jul 6;10(14):6993–7005. doi: 10.1002/ece3.6380 (PMC7391336; doi:10.1002/ece3.6380)
Supplement: Supplementary file 8 — Appendix S8 [file ECE3-10-6993-s008.pdf]

Supplementary Material 8: Added mass force ($F_M/\rho V$ of Eq (5)) depending on the acceleration of the strike (a in m.s^{-2}) for the five head models tested. Linear regression lines are drawn. Linear regression lines are drawn using dashed lines, the regression coefficients (y) correspond to the drag coefficient (C_a) of each shape and are indicated in the table below the graph. To compare with previous work (Segall *et al.*, 2019), the drag coefficients associated with the mean head shape of non-aquatically (orange line) and aquatically (dark blue line) foraging snakes have been added using solid lines.

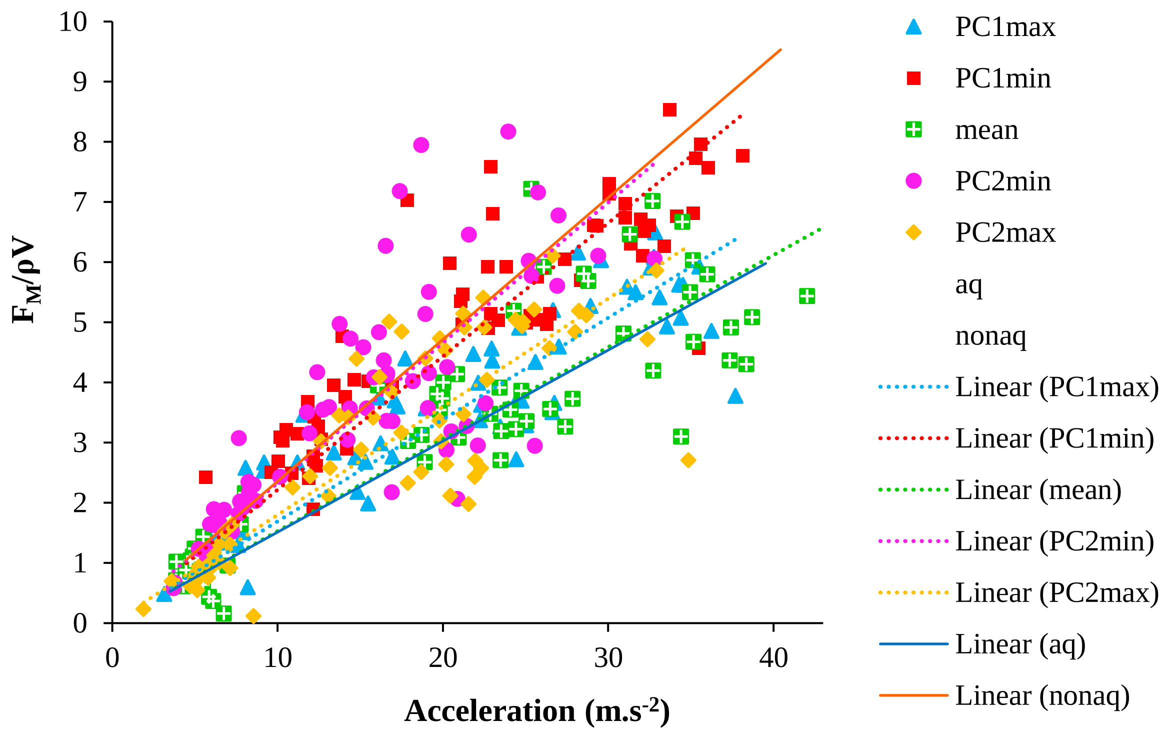

Model	C_a	R^2	N
PC1max	0.1692	0.7752	61
PC1min	0.2217	0.7263	65
Mean	0.1529	0.7021	61
PC2min	0.233	0.5579	65
PC2max	0.1798	0.7097	69
